# Students’ learning growth in mental addition and subtraction: Results from a learning progress monitoring approach

**DOI:** 10.3389/fpsyg.2022.944702

**Published:** 2022-11-28

**Authors:** Sven Anderson, Michael Schurig, Daniel Sommerhoff, Markus Gebhardt

**Affiliations:** ^1^Faculty of Rehabilitation Sciences, TU Dortmund University, Dortmund, Germany; ^2^Department of Mathematics Education, IPN – Leibniz Institute for Science and Mathematics Education, Kiel, Germany; ^3^Faculty of Human Sciences, University of Regensburg, Regensburg, Germany

**Keywords:** learning progress monitoring, mathematics education, mental computation, latent growth curve model, continuous norming, learning progression, formative assessment, curriculum-based measurement (CBM)

## Abstract

The purpose of this study was to measure and describe students’ learning development in mental computation of mixed addition and subtraction tasks up to 100. We used a learning progress monitoring (LPM) approach with multiple repeated measurements to examine the learning curves of second-and third-grade primary school students in mental computation over a period of 17 biweekly measurement intervals in the school year 2020/2021. Moreover, we investigated how homogeneous students’ learning curves were and how sociodemographic variables (gender, grade level, the assignment of special educational needs) affected students’ learning growth. Therefore, 348 German students from six schools and 20 classes (10.9% students with special educational needs) worked on systematically, but randomly mixed addition and subtraction tasks at regular intervals with an online LPM tool. We collected learning progress data for 12 measurement intervals during the survey period that was impacted by the COVID-19 pandemic. Technical results show that the employed LPM tool for mental computation met the criteria of LPM research stages 1 and 2. Focusing on the learning curves, results from latent growth curve modeling showed significant differences in the intercept and in the slope based on the background variables. The results illustrate that one-size-fits-all instruction is not appropriate, thus highlighting the value of LPM or other means that allow individualized, adaptive teaching. The study provides a first quantitative overview over the learning curves for mental computation in second and third grade. Furthermore, it offers a validated tool for the empirical analysis of learning curves regarding mental computation and strong reference data against which individual learning growth can be compared to identify students with unfavorable learning curves and provide targeted support as part of an adaptive, evidence-based teaching approach. Implications for further research and school practice are discussed.

## Introduction

Mental computation can be defined as a person’s ability to perform basic arithmetic operations correctly and quickly in their mind by using adequate solution strategies without resorting to external resources such as paper and pencil or a calculator (e.g., Maclellan, 2001; Varol and Farran, 2007). Focusing on current curricula, mental computation has an essential place in primary school mathematics education (e.g., the Standing Conference of the Ministers of Education and Cultural Affairs of the Länder in the Federal Republic of Germany (KMK), 2005; Seeley, 2005; National Council of Teachers of Mathematics (NCTM), 2022). This importance can be explained by the fact that mental computation has a high value in everyday life (e.g., Reys, 1984). Moreover, previous research has pointed to the great influence of mental computation for higher-order mathematical thinking (e.g., Blöte et al., 2000; Hickendorff et al., 2019; Pourdavood et al., 2020). In particular, mental computation can support students in understanding the concept of numbers, in discovering computational strategies, in making reasonable estimates and in developing a flexible and adaptive handling of these when solving mathematical problems. Furthermore, mental computation is a basis for written computation and its mastery.

Research findings indicate that most students improve their mental computation skills during primary school years and are able to solve multi-digit addition and subtraction tasks adequately in grades 3 or higher (e.g., Heirdsfield and Cooper, 2004; Karantzis, 2011). However, empirical research also shows that a large number of students struggle with mental computation throughout and beyond primary school (e.g., Reys et al., 1993; Miller et al., 2011; Hickendorff et al., 2019). Recent research findings (e.g., Peltenburg et al., 2012; Gebhardt et al., 2015; Rojo and Wakim, 2022) suggests, that the acquisition of multi-digit mental computation is particularly challenging for students with special educational needs (SEN). For example, students with SEN in the area of learning (SEN-L) mostly exhibit a lack of solid basic arithmetic skills, which is often responsible for difficulties and missing learning success in secondary school mathematics (e.g., Gebhardt et al., 2014; Rojo and Wakim, 2022). Studies focusing on the mathematical learning development of students with and without SEN conclude that students with SEN not only show a lower mathematical achievement, but also have a slower learning growth than their peers without SEN (e.g., Wei et al., 2013; Gebhardt et al., 2015).

In light of the importance of mental computation for further mathematical achievement and the high number of students who have difficulties developing adequate mental computation skills, there is great need for providing information about students’ learning growth in educational research and practice (e.g., Salaschek et al., 2014). For teachers in particular, summative assessments at the beginning or end of the school year are often insufficient in identifying struggling students at an early stage. An alternative are formative assessments, which provide diagnostic information during the learning process and allows for instructional adjustment (e.g., Cisterna and Gotwals, 2018). One formative approach is learning progress monitoring (LPM) which is discussed as an appropriate method to provide teachers with ongoing feedback on students’ learning development (e.g., Deno et al., 2009). To evaluate learning growth with LPM tools, teachers regularly administer short parallel tests and assess students’ individual learning curves using LPM graphs. The evaluation of these individual learning curves is the basis for decisions about maintaining or adjusting educational instructions. For example, within the Response to Intervention (RTI) approach, LPM tools are used to identify struggling students who would benefit from additive educational instruction or to evaluate the effectiveness of learning offers (e.g., Stecker et al., 2008).

In order to address the need for information on student learning development and learning growth in mental computation in educational research and practice, the purpose of the present study was to examine the latent learning curves as mean learning growth of the individual learning curves of second and third grade students in mental computation of mixed addition and subtraction tasks. Therefore, we used a recently developed computation test (Anderson et al., 2022). The present study first investigated the psychometric quality of this test for LPM. This included how the measures are related to student performance on standardized arithmetic tests and whether the LPM tool can sensitively measure student’s learning and progress at different ability levels. Subsequently, we used the data to describe students’ latent learning curves regarding mental computation skills in addition and subtraction over a period of 17 biweekly measurement intervals. Based on this, we examine differential developments using sociodemographic characteristics such as gender and grade level as well as the assignment of SEN.

## Mental addition and subtraction and its differential development

Mental computation skills regarding basic arithmetic are an important prerequisite for the acquisition of mathematical literacy as measured in international school performance studies such as the Programme for International Student Assessment (PISA; Organisation for Economic Co-operation and Development (OECD), 2018). Moreover, these competencies are inherent to the primary school mathematics curricula. In particular, mastering mental computation of multi-digit addition and subtraction tasks is an important learning goal in primary school all over the world. According to primary school mathematics curricula of all federal states in Germany or in the United States (e.g., KMK, 2005; NCTM, 2022), students should have developed profound mental addition and subtraction skills in the number range up to 100 by the end of grade 2. Based on a spiral approach, mental addition and subtraction skills are extended to three-digit numbers at the beginning of grade 3. By the end of primary school, students should be able to transfer these skills to higher number ranges. Subsequently, in the second half of grade 3, students learn the written algorithms for addition and subtraction of three-digit numbers (Selter, 2001). Considering the curricular requirements, third graders should therefore be able to routinely carry out two-digit mental addition and subtraction tasks whereas this may be more challenging for second graders.

The results of previous studies (e.g., Bryant et al., 2008; Karantzis, 2011) indicate that some students’ performance in mental addition and subtraction is at a low level even at higher grades, implying urgent need for educational means to address this issue. Weak performance in in this area in primary school is attributed to different task characteristics that contribute to task difficulty (e.g., Benz, 2003, 2005) and the use of inefficient solving strategies (e.g., Beishuizen, 1993; Cooper et al., 1996; Beishuizen et al., 1997; Heirdsfield and Cooper, 2004; Varol and Farran, 2007).

Regarding the task characteristics, the construction of the numbers, whose sum value or difference value needs to be calculated, plays an important role. Research has shown that multi-digit addition and subtraction tasks vary in their difficulty and probability of solving them correctly (e.g., Benz, 2003, 2005). This is explained by the fact that there are multiple difficulty-generating item characteristics (DGICs) that have an influence on task difficulty (e.g., the number of digits of a term or the necessity of crossing ten). Knowledge about the influence of different DGICs is particularly important for rule-based item design of school achievement tests (e.g., for statistical word problems see Holling et al., 2009). For mathematical word problems, Daroczy et al. (2015) provide a review of DGICs that contribute to the difficulty of such tasks. Anderson et al. (2022) discuss the advantages of rule-based item design and the identification of DGICs for constructing a pool of items for a mixed addition and subtraction test for LPM.

Besides that, the flexible and adequate use of different solution strategies for solving multi-digit addition and subtraction tasks is relevant (for an overview, e.g., Torbeyns et al., 2009; Hickendorff et al., 2019). While solving single-digit addition and subtraction tasks is based on the retrieval of the solution from long-term memory as an arithmetic fact, the outcome of multi-digit addition and subtraction tasks must be computed based on the adaptive application of known solution strategies. With the use of inefficient solution strategies such as counting strategies, multi-digit addition and subtraction tasks are solved slowly and often incorrectly. In addition to the flexibility in choosing appropriate solution strategies in correspondence with the requirement of a specific task, hurdles for struggling students include a lack of the conceptual understanding of numbers and a lack of fluency in using computation procedures (e.g., Verschaffel et al., 2007).

With regard to students’ solving strategies of multi-digit addition and subtraction tasks, two complementary dimensions can be distinguished respecting number-based strategies: the operation that is necessary for the solution process and the way the numbers are used in the solution process (Hickendorff et al., 2019). Concerning the first dimension, multi-digit addition only allows direct addition, while multi-digit subtraction allows several options (direct subtraction, indirect addition, indirect subtraction). Concerning the second dimension, there are different strategies to manipulate numbers to successfully master the computation process. In sequencing strategies, numbers are interpreted as objects on a mental number line and addition is seen as moving forward and subtraction as moving backward on it. For example, the addition task 44 + 38 is given. The direct addition with the sequencing strategy would be computed as 44 + 30 = 74; 74 + 8 = 82. In decomposition strategies, numbers are interpreted as objects with a decimal structure and the operations require splitting or portioning the numbers. With the decomposition strategy it would be computed as 40 + 30 = 70; 4 + 8 = 12; 70 + 12 = 82. In varying situations, different strategies are used that adaptively consider both the numbers and the operations in the solution process. These two complementary dimensions can be used to categorize students’ problem-solving strategies. Students with mathematical difficulties often have problems acquiring the different strategies and using them in an adaptive and flexible way. These students use inefficient solution strategies (e.g., counting strategies) and are often unable to accurately solve single-digit addition and subtraction, which is a prerequisite for successful acquisition of multi-digit strategy skills (e.g., Varol and Farran, 2007; Verschaffel et al., 2007).

Despite the high curricular importance, previous qualitative studies already indicate a large heterogeneity in the development of two-digit addition and subtraction computation skills during the second school year (e.g., Benz, 2003, 2007). Previous research has also shown that students with SEN have difficulty acquiring adequate computation skills (Gersten et al., 2005; Evans, 2007; Bryant et al., 2008; Wei et al., 2013; Soares et al., 2018). For example, at the end of primary school, many students with SEN-L have acquired lower competencies in the development in mathemtics in general (e.g., Gebhardt et al., 2014, 2015) and in the development of mental arithmetic computation for numbers up to 100 compared to their peers without SEN-L (Peltenburg et al., 2012; Rojo and Wakim, 2022).

While research findings on the difficulties for students with SEN-L are consistent, this is not the case for gender-based performance differences in mental computation (e.g., Winkelmann et al., 2008; Wei et al., 2013; Pina et al., 2021). Wei et al. (2013) reported significant and persistent gender performance differences in favor of boys among students with different SEN that persisted from primary to secondary school. For regular primary education, Pina et al. (2021) found no significant gender differences in mathematics achievement in computation. Results from international large-scale assessments such as the Trends in International Mathematics and Science Study (TIMSS) indicated gender-based differences in average mathematics achievement between girls and boys at the end of primary school. In TIMSS 2019, fourth grade boys showed a higher average performance than girls in almost half of the 58 participating countries. In four countries, girls had a higher average achievement than boys. In 27 countries, gender equity of average performance in mathematics was reported. For the arithmetic domain, boys achieved higher test scores than girls in almost all countries and for more than half of the countries’ differences are even significant (Mullis et al., 2020). In Germany, the differences in this domain are significant (Nonte et al., 2020).

In contrast, in a study of third-through eighth-graders with and without SEN, Yarbrough et al. (2017) found statistically significant differences between boys and girls in favor of girls in grades 5, 7, and 8 for learning growth in mental computation. The tests included mathematical computation tasks on the four basic arithmetic operations. The difficulty of the tasks varied according to the respective curricular requirements of the respective grade. However, the knowledge about students’ differential latent learning curves when acquiring mental computation skills is limited. For example, the results on learning growth by Yarbrough et al. (2017) were based on only three measurement time points. As noted above, there is only a small number of longitudinal surveys, including a large number of measures for the valid assessment of latent learning curves.

## Learning progress monitoring

Due to heterogeneous student learning, there is an increasing need for teachers to use data about individual student’s learning development for their instructional decision-making (e.g., Espin et al., 2017). In this regard, LPM is a promising method that provides data on individual students’ learning development and assists teachers identifying learning problems in early stages as well as in evaluating the achievement of learning goals. One approach of LPM is curriculum-based measurement (CBM; e.g., Deno, 1985; Stecker et al., 2005): a set of procedures that can be used frequently and quickly to assess student learning progress and the effectiveness of instruction in academic domains such as reading, spelling, writing, or computation (e.g., Hosp et al., 2016). CBM procedures consist of short parallel tests that require only a few minutes (e.g., 1–5 min) and items are typically based on the identification of robust indicators or on curriculum sampling (Fuchs, 2004). Finding robust indicators includes identifying tasks that best represent the various subskills of a specific domain or that correlate strongly with them. For reading, oral reading fluency is regarded as a robust indicator of general reading competence and comprehension (Deno et al., 1982). In the domain of mathematics, number sense is considered a robust indicator for mathematics performance in kindergarten and first grade primary school (e.g., Lembke and Foegen, 2009). Curriculum sampling involves selecting exemplary tasks that asses curricular learning goals. Each CBM test is then aligned with curricular objectives that are relevant to the entire assessment period (e.g., Fuchs, 2004). With regard to highly heterogeneous learning groups, for example in inclusive classrooms, strictly curriculum-based LPM are of limited use because students with SEN (e.g., SEN-L) are often not taught according to the regular class curriculum (Gebhardt et al., 2016).

Results of LPM usually output a sum score (e.g., number of correctly solved tasks) and the learning development is represented in a graph. To represent individual learning development, linear trends at the student level are often estimated. Therefore, the parameters intercept and slope are relevant. The slope represents the mean learning growth of a student (e.g., the proportion of additional tasks that were solved correctly in the comparison of the measurement points). The intercept contains information about the approximated individual learning level at the beginning of LPM. For reliable and valid conclusions about learning development, Christ et al. (2013) recommend using data from at least six measurements. Based on these data, teachers can then decide whether the instruction used promotes learning success as intended (individual learning curve is as expected), whether the instruction used should be adjusted (individual learning curve is lower than expected), or whether the learning goal can be adjusted because the individual learning curve is higher than initially expected (e.g., Espin et al., 2017).

Since the 1970s, a large body of LPM research has focused on the development and application of instruments for different domains with a focus on reading (for an overview see Tindal, 2013). Until 2008, LPM research mostly focused on the domain of reading and not mathematics (e.g., Van Der Heyden and Burns, 2005; Foegen et al., 2007). In a review of the literature concentrating on the development of LPM in mathematics, Foegen et al. (2007) found that only a small part of the studies focused on mathematics and here primarily on preschool and elementary mathematics. In German-speaking countries, LPM research has advanced in recent years, especially in educational psychology and special education, addressing several academic domains, including reading and math (for an overview see Breitenbach, 2020; Gebhardt et al., 2021).

### Learning progress monitoring of mathematics computation

Regarding different types of LPM in mathematics, Hosp et al. (2016) differentiate between tests for number sense (early numeracy), for computational skills (computation), and for the application of mathematical skills such as interpreting measurements, tables, or graphs (concepts and applications). For LPM in the area of computational skills, there are differences in how the tasks are intended to be solved (e.g., in a mental or written way) and how large the assessment domain is in each case (assessment of a single skill or multiple skills). According to Christ et al. (2008), the domain of mathematics computation is especially suitable for a frequently used LPM tool that can be used in research as well as data-based instructional decision-making. Instruments for this domain are usually constructed to provide very brief measurements of a relatively narrow arithmetic performance range and LPM tasks corresponding to the curricular level or individual learning objectives. There is also evidence that teachers can use the data of computation LPM to improve the performance of students with SEN (for an overview see Foegen et al., 2007).

LPM of (mental) computational skills does not aim to measure mathematical literacy as in PISA (OECD, 2018), and it does not address the language requirements of number word problems, which can also play a role in understanding mathematics. In contrast, it focuses on (mental) computation skills as an important prerequisite for solving word problems as well as mathematical literacy in general (e.g., Varol and Farran, 2007). Still, this narrow focus must be considered when selecting potential criterion measures for the evaluation of criterion validity. According to Christ et al. (2008), the coefficients of criterion validity between LPM tools and standardized mathematical achievement tests that measure overall performance in mathematics can therefore only be interpreted to a limited extent, whereas criterion validity is understandably much higher for procedures that relate exclusively to arithmetic tasks or include subtests in the domain of computation.

A variety of LPM tools for computation and mental computation have been developed in the past decades, especially in the United States (for an overview, e.g., Christ et al., 2008; Tindal, 2013). In German-speaking countries, some tools have been established for LPM (mental) computation. For example, Sikora and Voß (2017) have developed and empirically validated a curriculum-based LPM tool for the four basic arithmetic skills for grades 3 and 4. In composing the LPM tests, they considered item characteristics that may influence item difficulty (e.g., number range, arithmetic operation, digits to be computed in item solution, place value, and standard form tasks). The LPM tests of Strathmann and Klauer (2012) or Salaschek and Souvignier (2014) have integrated mental computation tasks as part of a broader curriculum-based LPM tool. These are usually a subset of a few items, each testing one of the four basic arithmetic competencies at the respective curricular level. Anderson et al. (2022) developed a test based on an item-generating system for mixed addition and subtraction tasks for numbers up to 100. This test is built on multiple difficulty-generating item characteristics (DGICs). First, three DGICs were deduced from prior mathematics education research (arithmetic operation, necessity of crossing ten, the number of second term digits) and varied within the item design process so that all possible combinations were adequately represented in an item pool. Subsequently the Rasch model (RM) and the Linear Logistic Test Model (LLTM) were used to estimate and predict the influence of the DGICs. The results of the LLTM approach indicate that all three suspected difficulty-generating characteristics were significant predictors of item difficulty and explain about 20% of the variance in the item difficulty parameters of the RM. Results suggest that DGICs can influence item difficulty across grade levels and ensure long-term use across multiple grade levels. Thus, identified curriculum-independent DGICs have the potential to be used to construct LPM tests for classes with curriculum-independent learners. In test development, the present study follows the item generation system reported by Anderson et al. (2022) and extends it to include an additional DGIC.

### Requirements for learning progress monitoring

In order to validly assess learning progress, frequent and regular use of LPM requires a large number of parallel tests that should be mostly consistent in difficulty and are sufficiently sensitive to measure learning. Therefore, LPM tools have to address a variety of psychometric properties. This includes classical test quality criteria (e.g., validity, reliability) as well as psychometric criteria such as one-dimensionality, homogeneous test difficulty, sensitivity to change, and test fairness (e.g., Wilbert and Linnemann, 2011; Schurig et al., 2021). For example, identifying characteristics that have an influence on task difficulty can support the development of parallel tests with homogeneous test difficulty (e.g., Wilbert, 2014; Anderson et al., 2022). In this regard, LPM tests should be constructed under the assumptions of item response theory (IRT), which features sample independence, non-linear dependencies between trait and response, and the ability to test multiple parameters of response behavior (e.g., Schurig et al., 2021). For the practical purpose of data-based decision-making, the results should also be as easy as possible for teachers to interpret and use to choose or adapt instruction (e.g., Espin et al., 2017). In particular, computer-or web-based LPM tools can contribute to improving the usability in schools through a high degree of automation of test generation and evaluation (e.g., Mühling et al., 2019).

As evidence for its use in progress measurement, Fuchs (2004) proposed a three-stage systematization of LPM research. Research at stage 1 includes studies that aim to test the psychometric adequacy of the tool as a status diagnostic. Stage 2 includes all research that provides evidence that a LPM tool can sensitively and validly represent learning growth over time. Research at stage 3 involves studies that examine whether the use of LPM data for instructional decisions improves student performance. For all academic domains, a large part of the prior research has focused on stage 1 and adressed the psychometric adequacy of LPM tool as a status diagnostic (Fuchs, 2017).

## Purpose of the study

The purpose of the present study was to examine the latent learning curves of second and third grade primary school students in mental addition and subtraction with a newly developed web-based LPM tool. Our study thus addresses stages 1 and 2 outlined by Fuchs (2004). Therefore, we examined the psychometrical adequacy of the LPM tool at an individual measurement point as well as its sensitivity to learning growth over time by addressing the following research questions:

Research question 1.1: How do the LPM test scores at different measurement time points relate to standardized school achievement test results at the beginning and the end of the survey period?
Research question 1.2: How reliable are the results of our LPM tool in terms of correlations between different measurement time points?
Research question 1.3: Is the LPM tool sensitive to student learning at different ability levels?

Building on these analyses, we subsequently examined students’ latent learning curves regarding mental addition and subtraction in second and third grade over a period of 17 biweekly measurement intervals, focusing on the overall learning development over time as well as interindividual heterogeneity therein. As prior results have highlighted that sociodemographic characteristics can influence learning and learning development, we additionally examined, if gender and grade level as central sociodemographic characteristics as well as the assignment of SEN lead to empirically distinguishable learning curves. Research questions are as follows:

Research question 2.1: How homogeneous are students’ latent learning curves over a period of 17 biweekly measurement intervals?
Research question 2.2: Do students’ latent learning curves differ between groups with different sociodemographic characteristics such as gender and grade level?
Research question 2.3: What influence does the assignment of SEN have on students’ latent learning curves in mental computation?

## Materials and methods

### Participants and setting

A total of 348 students from nine second-grade and nine third-grade inclusive education classes and two third-grade special education classes^1^ of six schools participated in the study. The schools were located in urban as well as rural areas of North Rhine-Westphalia as state of the Federal Republic of Germany. The six schools were recruited by convenience sampling. Therefore, it was taken into account that a similar number of second and third graders participated in the survey, as well as students in a special school and students in inclusive schools. Of the participating students, 162 (46.55%) were in the second grade, 186 (53.45%) in the third grade. The average age of students at the start of the study was 8.43 years (*SD* = 0.80). Further sociodemographic characteristics of the participating students at the start of the study are reported in Table 1. A number of 38 students (10.92%) had the assignment of SEN, most of them in the area of learning (SEN-L: 19; 50.00% of the SEN students) or in the area of communication and interaction (SEN-CI: 17; 44.74% of the SEN students).

**Table 1:** Sociodemographic of students at the start of the study.

Personal characteristics	Full sample		Grade 2		Grade 3	
	*n*	%	*n*	%	*n*	%
Gender						
Female	176	50.57	85	52.47	91	48.92
Male	172	49.43	77	47.53	95	51.08
SEN						
Yes	38	10.92	2	1.23	36	19.35
No	310	89.08	160	98.77	150	80.65
Migration background						
Yes	96	27.59	46	71.60	50	26.88
No	252	72.41	116	28.40	136	73.12

### Measures and procedure

The study was conducted from November 2020 to July 2021 and covered a period of 17 biweekly measurement intervals. At the beginning and at the end of the survey period, arithmetic subscales of the standardized German paper-pencil test DEMAT 2+ (German Mathematics Test for Second Grade and for the beginning of Third Grade; Krajewski et al., 2020) were administered. The DEMAT 2+ is representative of all German regular second-grade mathematics curricula and is suitable as a norm-based test for the last months of the second and the first months of the third school year. The test contains tasks for numbers up to 100. For this study, we selected subscales of the DEMAT 2+ that included computation tasks without mathematics word problems. These included tasks for number properties, addition and subtraction place values tasks, tasks for doubling and halving numbers, and tasks for calculating with money (see Table 2). The use of DEMAT 2+ subscales at the beginning were followed by LPM every 2 weeks. At the end of the survey period, DEMAT 2+ subscales were administered a second time. Credit was given only for completely correct answers.

**Table 2:** Subscales of the DEMAT 2+ (Krajewski et al., 2020) used for this study.

Subscale DEMAT 2+	Requirement	Example	No. of items
Number properties	Identification of even and odd two-digit numbers	Identify the even numbers! 25|44|8|19|8|38|17	2
Addition place value	Identification of the correct first/second summand	Calculate! … + 15 = 34	4
Subtraction place value	Identification of the correct subtrahend/minuend	Calculate! 56 - … = 36	4
Doubling numbers	Doubling of a two-digit number (with and without crossing ten)	Take the double! 70 ➔ …	3
Halving numbers	Halving of a two-digit number (with and without crossing ten)	Take the half! 24 ➔ …	3
Calculating with money	Calculation of a two-digit cent amount to get 1 € (1 € = 100 cents)	How many cents are missing if you want 1€? At 45 cents missing …	4

The used LPM tool included mixed addition and subtraction tasks for numbers up to 100, which required students to enter the correct solution (for addition tasks the sum value; for subtraction tasks the difference value) into a blank field. We designed the items using a rule-based approach that considered several DGICs derived in advance from mathematics education research and evaluated in Anderson et al. (2022). Extending the results of Anderson et al. (2022) four DGICs were used to model the difficulty of the items: the arithmetic operation (addition versus subtraction; DGIC 1), the necessity of crossing ten (no crossing versus with crossing; DGIC 2), the number of second term digits (one-digit numbers versus two-digit numbers; DGIC 3), and the necessity to add up to the next full ten (not necessary versus necessary; DGIC 4). Based on these four DGICs, we created a pool of 3,027 items. The four DGICs were varied within the item design process so that all possible combinations were adequately represented in the item pool (see Table 3).

**Table 3:** Sample items illustrating different types of items based on the four DGICs.

Category	Example	DGIC 1	DGIC 2	DGIC 3	DGIC 4
1	27 + 2	Addition	No	One	No
2	23 + 13	Addition	No	Two	No
3	21 + 9	Addition	No	One	Yes
4	52 + 38	Addition	No	Two	Yes
5	78 + 9	Addition	Yes	One	No
6	67 + 27	Addition	Yes	Two	No
7	48–3	Subtraction	No	One	No
8	98–24	Subtraction	No	Two	No
9	65–7	Subtraction	Yes	One	No
10	91–16	Subtraction	Yes	Two	No

DGIC 1: Arithmetic operation; DGIC 2: Crossing ten; DGIC 3: No. of 2nd term digits; DGIC 4: Add up to the next full ten.

The item pool was implemented on an online platform^2^ (Gebhardt et al., 2016; Mühling et al., 2019). Based on an equal distribution of the 10 possible item categories in the item selection, a fixed order was established for the baseline test. Starting at the second measurement, items were drawn from the total item pool in a randomized, however equally distributed manner according to the 10 item categories. Students could not skip any drawn items during the test time. We assume that an equal distribution of the items on the described item categories causes a harmonization of the difficulties of the tests. Based on this, an individual test was created for each student by the online platform for each additional measurement. Accordingly, from the second measurement on, we assume missing completely at random (MCAR) for all non-drawn items.

Trained administrators tested students in their classrooms in groups of 5–10 during class time. To perform the test, each participating student used a tablet device. Testing time was 5 min. The students had to mentally compute the tasks without external support. At the beginning of each measurement, students received a short technical briefing, sample tasks were solved, and students had the opportunity to ask the test administrator questions. Students could then start the test themselves by clicking on a start button. Tests ended automatically after 5 min testing time. In the time allotted, students were instructed to answer as many mathematics computation tasks as possible. Each probe contains a substantial number of tasks, making it unlikely that a student could finish within the time limit. No partial credit was given for partially correct answers.

All students who participated in at least one LPM test were included in the following analyses. Not all students participated in LPM at each measurement. The main reasons for this were home schooling periods during the survey due to the COVID-19 pandemic, a staggered start to the surveys within the participating schools, individual absence of students, or technical problems. In order to compute the latent mean-growth and comparable, time-dependent norms across the survey time, 17 equidistant measurement intervals were derived from the raw data. In order to establish reasonable distance interval lengths to observe change, 2 weeks were chosen as the length of the interval. Due to practical reasons within the schools, some children were tested twice within one interval and not within another. When students were tested twice, only the first observation within a measurement interval was used. Data are available for 12 of the 17 biweekly measurement intervals, the other intervals are missing due to homeschooling and holidays.

### Statistical analyses

#### Participation

The individual number of participation related to the LPM measurement intervals in this study varied (*M* = 5.98; *SD* = 2.20). The range of participation is 10, with 24 students (6.90%) of the total sample participating in the surveys only once and 5 students (1.44%) participating 11 times. 282 students (81.03%) participated in at least five, 222 students (63.79%) in at least six LPM measurement intervals.

#### Analyses

The presentation of descriptive statistics is followed by the results on the research questions. To address research question 1.1, criterion and predictive validity were analyzed by examining how LPM scores relate to the employed arithmetic subscale scores of the standardized paper-pencil mathematics test DEMAT 2+. To answer research question 1.2, a RM was fitted for every single LPM test. Due to the high number of missing data by design, the item fit was evaluated using a conditional pairwise item category comparison implemented in the *R* package *pairwise* (Heine and Tarnai, 2015). The pairwise approach is able to handle (completely) random missing data by design. Subsequently, alternate form test–retest reliability for adjacent and more distant tests was calculated. Regarding research question 1.3, performance is assessed by the continuous norming method using the *R* package *cNorm* (Lenhard et al., 2018) to evaluate how sensitive the test measures at different ability levels at different measurement intervals. In *cNorm*, norm values and percentiles are estimated as a function of time and possibly covariates using Taylor polynomials. To identify adequate test norms, a polynomial regression model needs to be found that describes the norming sample as accurately as possible with the minimum number of predictors. Lenhard and Lenhard (2021) emphasized that higher numbers of terms do often lead to overfit. Therefore, *cNorm* used 𝑘 = 4 terms by default. In the modeling process the stopping criterion is 𝑅^2^ = 0.99.

In our case, the explanatory variable represents the different measurement intervals over the LPM survey period of 17 biweekly measurement intervals Thus, the *cNorm* approach addresses some disadvantages of traditional norming methods such as a high sample size, the consideration of sampling errors or any distributional assumptions. Moreover, gaps between discrete levels of the explanatory variable can be closed (Gary et al., 2021). This can be particularly advantageous for LPM, since norm tables can be generated not only for the discrete measurement point of the survey, but also for each subsequent measurement point, even if no measurement has occurred. In our case, this means that norm values could also be derived for the measurement intervals where no LPM tests were conducted due to homeschooling.

In order to address research questions 2.1–2.3, latent learning curves and the modeling of individual differences in learning growth over time including sociodemographic characteristics such as gender or grade and assignment of SEN are examined *via* latent growth curve modeling (LGCM; e.g., Muthen and Khoo, 1998). In educational and psychological contexts, this approach is often used to determine learning growth and the influence of background variables in LPM longitudinal data (e.g., Salaschek and Souvignier, 2014; Johnson et al., 2020). The lavaan package (Rosseel, 2012) in was used to estimate latent growth curve models.

**Figure 1:**
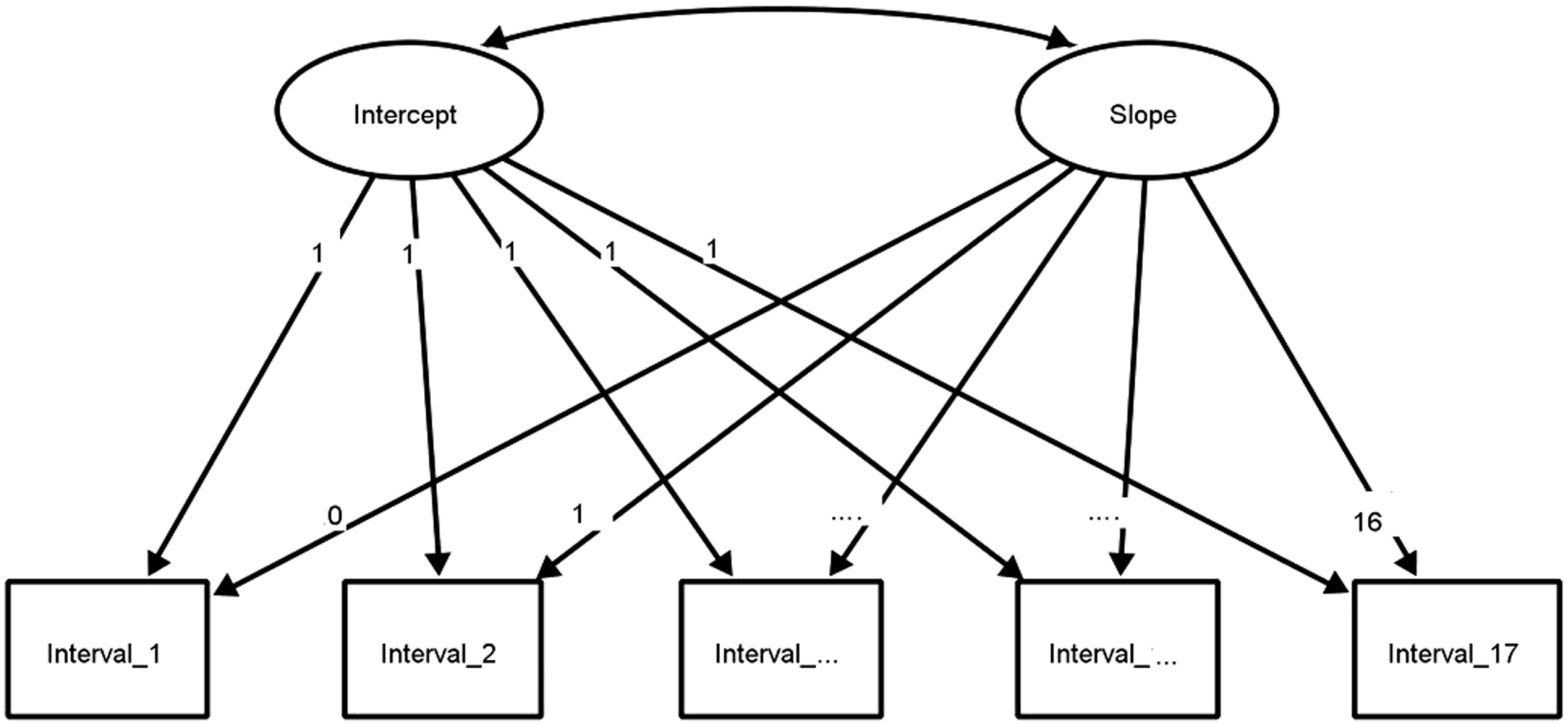
Graphical representation of a LGCM for 17 measurement intervals without covariates. The three dots represent the intervals 3–15, which were not included in this representation for greater clarity. The graphical representation of the growth model was created with Ωnyx (von Oertzen et al., 2015).

The LGCM illustrates the use of slope and intercept as two latent variables to model differences over time. The student’s initial performance in solving mixed addition and subtraction tasks for numbers up to 100 is represented as a scale score (intercept). Similarly, the rate of linear growth in the student’s competences across all measurement intervals is represented as a scale score (slope). The initial LGCM (Model 1) represented in Figure 1, includes each biweekly administration of LPM, except for measurement intervals 4–7 and 11 when no measurements could be taken in schools due to the COVID-19 pandemic and the switch to home schooling. Furthermore, it was analyzed if sociodemographic variables such as gender or grade level or the assignment of SEN influence learning growth. For this, the LGCM was extended to include group differences (Model 2). We used gender (0 = male, 1 = female), grade level (0 = grade 2, 1 = grade 3), and special educational need (0 = no, 1 = yes) as dummy coded variables across the 12 measurement intervals. In Model 2, the intercept and slope variables are predicted while considering these background variables.

## Results

Descriptive statistics for the LPM tests at each of the 12 measurement intervals are presented in Table 4 for the full sample and separately for both grade levels. With regard to the measurement intervals 1–3 and 14–17, regular mathematics instruction took place at school. In contrast, the measurement intervals in between were often characterized by home schooling due to the COVID-19 pandemic when mathematical instruction often took place *via* distance learning and not all students were able to regularly participate in LPM testing.

**Table 4:** Descriptive statistics of LPM scores for each measurement interval.

Time of measurement	Full sample		Grade 2		Grade 3	
	*n*	*M* (*SD*)	*n*	*M* (*SD*)	*n*	*M* (*SD*)
Measurement interval 1	194	11.01 (7.80)	85	5.62 (4.00)	109	15.20 (7.46)
Measurement interval 2	256	10.24 (8.09)	131	6.00 (5.28)	125	14.69 (8.15)
Measurement interval 3	108	13.46 (9.46)	43	7.79 (6.59)	65	17.22 (9.23)
[…]	[…]	[…]	[…]	[…]	[…]	[…]
Measurement interval 8	150	12.21 (9.30)	89	9.47 (8.01)	61	16.20 (9.66)
Measurement interval 9	225	13.30 (10.02)	109	9.03 (7.91)	116	17.32 (10.17)
Measurement interval 10	152	15.11 (10.38)	81	11.27 (8.20)	71	19.49 (10.92)
[…]	[…]	[…]	[…]	[…]	[…]	[…]
Measurement interval 12	43	16.16 (11.27)	31	13.52 (10.33)	12	23.00 (11.10)
Measurement interval 13	46	16.91 (11.96)	34	13.56 (10.16)	12	26.42 (11.91)
Measurement interval 14	232	15.44 (9.46)	88	11.19 (7.90)	144	18.04 (9.41)
Measurement interval 15	291	15.65 (9.99)	133	11.97 (8.42)	158	18.75 (10.18)
Measurement interval 16	275	15.65 (10.26)	108	10.68 (6.65)	167	18.87 (10.90)
Measurement interval 17	107	17.42 (10.26)	59	14.59 (7.65)	48	20.90 (11.95)

LPM tests were canceled due to the COVID-19 pandemic in measurement intervals 4–7 and 11. Square brackets with three dots represent the canceled measurement intervals.

### Research question 1.1: Criterion validity

For reliability analysis, Cronbach’s alpha and Mc Donald’s omega were calculated to assess the internal consistency of the subscales of the selected DEMAT 2+ at the first and last measurement time point separately for grades 2 and 3 and the full sample. The internal consistency of the subscales of the DEMAT 2+ are satisfactory (see Tables 5, 6). Correlations of LPM sum scores with the overall sum scores of the subscales of the DEMAT 2+ at the first measurement time point were strong with a mean correlation of *r* = 0.73 (95%CI [0.68; 0.78]). For the full sample, the correlations of the various subscales of the DEMAT 2+ ranged from 0.39 (subscale number properties) to 0.67 (subscale calculating with money) with *M* = 0.57 and *SD* = 0.10.

**Table 5:** Cronbach’s Alpha and Mc Donald’s Omega coefficients at first measurement time point.

DEMAT 2+ subscale	Full sample		Grade 2		Grade 3	
	*α*	*ω*	*α*	*ω*	*α*	*ω*
Number properties (2 items)	0.78	0.78	0.78	0.78	0.77	0.77
Addition place value (4 items)	0.80	0.80	0.71	0.73	0.76	0.77
Subtraction place value (4 items)	0.73	0.74	0.70	0.71	0.69	0.70
Doubling numbers (3 items)	0.88	0.89	0.81	0.83	0.90	0.91
Halving numbers (3 items)	0.72	0.73	0.62	0.65	0.72	0.73
Calculating w. money (4 items)	0.90	0.90	0.86	0.87	0.89	0.89

*α* = Cronbach’s Alpha; *ω* = Mc Donald’s Omega; Full sample = 328 students (grade 2 = 155; grade 3 = 173).

**Table 6:** Cronbach’s Alpha and Mc Donald’s Omega coefficients at last measurement time point.

DEMAT 2+ subscale	Full sample		Grade 2		Grade 3	
	*α*	*ω*	*α*	*ω*	*α*	*ω*
Number properties (2 items)	0.76	0.76	0.66	0.66	0.82	0.82
Addition place value (4 items)	0.80	0.80	0.73	0.74	0.78	0.78
Subtraction place value (4 items)	0.61	0.62	0.57	0.58	0.58	0.63
Doubling numbers (3 items)	0.84	0.86	0.82	0.85	0.86	0.88
Halving numbers (3 items)	0.78	0.80	0.74	0.75	0.80	0.83
Calculating w. money (4 items)	0.89	0.89	0.86	0.86	0.89	0.89

*α* = Cronbach’s Alpha; *ω* = Mc Donald’s Omega; Full sample = 302 students (grade 2 = 130; grade 3 = 172).

At the last measurement time point, correlations of LPM sum scores with the overall sum scores of the DEMAT 2+ subscales were moderate with a mean correlation of *r* = 0.57 (95%CI [0.49; 0.64]). For the full sample, the correlations of the various DEMAT 2+ subscales ranged from 0.22 (subscale number properties) to 0.57 (subscale addition place value) with *M* = 0.42 and *SD* = 0.12 (for further information separately by grade level see Table 7).

**Table 7:** Correlations of LPM scores at the beginning and end of the survey with subscales of the DEMAT 2+.

Variables of DEMAT 2+	Full sample		Grade 2		Grade 3	
	LPM Begin.	LPM End	LPM Begin.	LPM End	LPM Begin.	LPM End
Beginning of survey						
Number properties	0.39**	0.23**	0.27**	0.10	0.41**	0.22**
Addition place value	0.63**	0.47**	0.49**	0.28**	0.53**	0.43**
Subtraction place value	0.57**	0.52**	0.38**	0.37**	0.56**	0.51**
Doubling numbers	0.54**	0.42**	0.37**	0.40**	0.54**	0.34**
Halving numbers	0.63**	0.45**	0.44**	0.34**	0.62**	0.41**
Calculation w. money	0.67**	0.53**	0.46**	0.40**	0.63**	0.48**
**Overall sum score subscales**	0.73**	0.56**	0.55**	0.44**	0.70**	0.53**
End of survey						
Number properties	0.36**	0.22**	0.22*	0.10	0.41**	0.24**
Addition place value	0.58**	0.57**	0.22*	0.38**	0.60**	0.60**
Subtraction place value^a^	0.47**	0.45**	0.28**	0.39**	0.47**	0.43**
Doubling numbers	0.36**	0.33**	0.28**	0.33**	0.39**	0.31**
Halving numbers	0.52**	0.47**	0.30**	0.32**	0.61**	0.51**
Calculation w. money	0.52**	0.47**	0.35**	0.44**	0.52**	0.42**
**Overall sum score subscales**	0.63**	0.57**	0.40**	0.49**	0.66**	0.55**

* indicates *p* < 0.5. ** indicates *p* < 0.01.

a The subscale did not reach an acceptable internal consistency (see Table 6).

To test the predictive validity of LPM measures, the correlation of the LPM sum scores at the first measurement time point with the overall sum scores of the DEMAT 2+ subscales at the last measurement time point were calculated. Correlations were moderate to strong with a mean correlation of *r* = 0.63 (95%CI [0.56; 0.70]; for grade 2: *r =* 0.40, for grade 3: *r* = 0.66). Correlations of DEMAT 2+ sum scores at the first and at the last measurement time point were strong with a mean correlation of *r* = 0.80 (95%CI [0.75; 0.84]; for grade 2: *r* = 0.61, for grade 3: *r* = 0.86).

### Research question 1.2: Reliability

The reliability of the resulting Weighted Maximum Likelihood Estimation (WLE) person parameters ranged from 0.80 to 0.85 (*M* = 0.82; *SD* = 0.02) for the measurement intervals. Furthermore, the alternate form test–retest reliability was calculated for each pair of adjacent and more distant tests (e.g., LPM interval 1 scores to LPM interval 2 scores, LPM interval 1 scores to LPM interval 17 scores, …, LPM interval scores 16 to LPM interval scores 17; see Table 8). Correlation indices between scores from adjacent measurement intervals ranged from 0.73 to 0.93. With reference to the COTAN review system for evaluating test quality (Evers et al., 2015), we interpret this as sufficient alternate form test–retest reliability.

**Table 8:** Correlations of LPM sum scores.

	MI 1	MI 2	MI 3	[…]	MI 8	MI 9	MI 10	[…]	MI 12	MI 13	MI 14	MI 15	MI 16
MI 1													
													
MI 2	0.80***												
	[0.73, 0.86]												
MI 3	0.83***	0.86***											
	[0.71, 0.90]	[0.79, 0.91]											
[…]													
MI 8	0.83***	0.85***	0.89***										
	[0.67, 0.91]	[0.79, 0.89]	[0.82, 0.93]										
MI 9	0.77***	0.78***	0.79***		0.84***		.						
	[0.67, 0.84]	[0.72, 0.83]	[0.68, 0.86]		[0.79, 0.89]								
MI 10	0.89***	0.85***	0.86***		0.87***	0.83***							
	[0.78, 0.94]	[0.79, 0.89]	[0.77, 0.91]		[0.82,0.91]	[0.76, 0.87]							
[…]													
MI 12		0.87***	0.89***		0.93***	0.95***	0.90***						
		[0.77, 0.93]	[0.77, 0.95]		[87, 0.96]	[0.90, 0.97]	[0.82, 0.95]						
MI 13		0.86***	0.88***		0.86***	0.90***	0.88***		0.87***				
		[0.74, 0.92]	[0.75, 0.94]		[0.75,0.92]	[0.82, 0.94]	[0.79, 93]		[0.77, 0.93]				
MI 14	0.76***	0.80***	0.79***		0.84***	0.86***	0.90***		0.86***	0.87***			
	[0.68, 0.82]	[0.73, 0.85]	[0.70, 0.86]		[0.76, 0.89]	[0.81, 0.89]	[0.84,0.93]		[0.76, 0.93]	[0.77,0.93]			
MI 15	0.66***	0.74***	0.72***		0.82***	0.78***	0.86***		0.91***	0.87***	0.86***		
	[0.56, 0.74]	[0.68, 0.79]	[0.60, 0.80]		[0.76, 87]	[0.72, 0.83]	[0.81, 0.90]		[0.83, 0.95]	[0.78, 0.93]	[0.82, 0.89]		
MI 16	0.70***	0.70***	0.76***		0.74***	0.71***	0.76***		0.94***	0.88***	0.77***	0.81***	
	[0.61, 0.77]	[0.63, 0.76]	[0.65, 0.84]		[0.64, 0.81]	[0.63, 0.78]	[0.67, 0.83]		[0.75, 0.99]	[0.60, 0.97]	[0.70, 0.82]	[0.76, 0.85]	
MI 17	0.64**	0.79***	0.74***		0.74***	0.70***	0.85***		0.91***	0.85***	0.81***	0.85***	0.81***
	[0.29, 0.84]	[0.71, 0.86]	[0.49, 0.87]		[0.64, 0.82]	[0.59, 79]	[0.79, 0.90]		[0.67, 0.98]	[0.54, 0.96]	[0.67, 0.90]	[0.78, 0.89]	[0.73, 0.87]

MI is the acronym for the term measurement interval. LPM tests were canceled due to the COVID-19 pandemic in measurement intervals 4–7 and 11. Values in square brackets indicate the 95% confidence interval for each correlation. Square brackets with three dots represent the canceled measurement intervals.*indicates *p* < 0.05; **indicates *p* < 0.01; ***indicates *p* < 0.001. The correlation of MI 1 to M 12 and MI 1 to MI 13 cannot be reliably calculated due to a low sample size.

### Research question 1.3: Generating continuous tests norms

**Figure 2:**
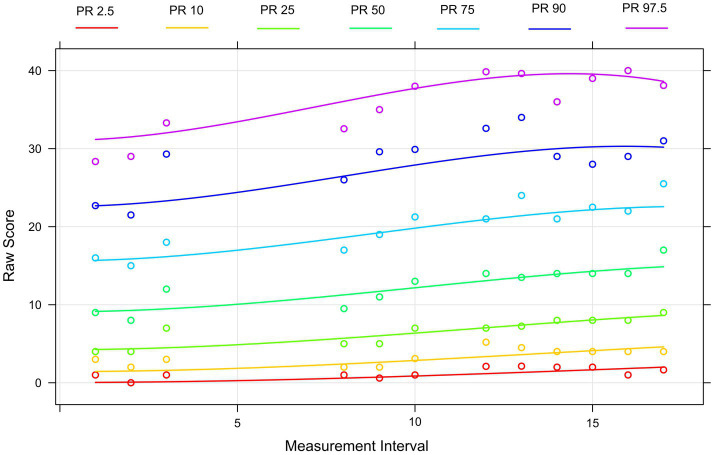
Percentile curves based on the sample of the mixed addition and subtraction LPM test. The curves show, which raw score (*y*-axis) is assigned to a specific ability level (each represented by a percentile curve) at a certain LPM measurement interval (*x*-axis).

As mentioned above, the procedure is robust to different or small sample sizes. The modeling procedure of the LPM scores from interval 1 to interval 17 reached an adjusted *R*^2^ = 0.98 with 5 terms and an intercept. It must be taken into account that at five measurement intervals no data collection could be conducted due to homeschooling and therefore *R*^2^ = 0.99 was not reached. To achieve this value, the number of terms would have to be increased further, which we have refrained to avoid an overfit. The norms in the upper range vary strongly. Figure 2 shows the assignment of the raw test values at the various levels to a specific percentile. Students with high raw scores at the beginning also have a higher slope over the survey period. The clustering of percentiles in the lower ranges (roughly up to the 25th percentile) do indicate a low separability. In other words: the test is probably still too difficult for as many as 25% of the students.

### Research questions 2.1–2.3: Sensitivity to learning

The investigation of students’ latent learning curves in mental addition and subtraction is presented in two steps. In a first step, we report the model fit of Model 1 (model without covariates) and Model 2. In a second step, we evaluate the latent learning curves regarding each of the research questions 2.1–2.3.

To estimate the model fit, we used the chi-square test, the root mean square error of approximation (RMSEA), the Tucker-Lewis Index (TLI), the Comparative fit index (CFI), and the standardized root mean square residual (SRMR). TLI and CFI values close to 0.95 indicate an adequate fit to the data. RMSEA values close to 0.06 and SRMR values close to 0.08 generally are recommended (Hu and Bentler, 1999).

A first LGCM was estimated (Model 1) to investigate the changes in the means of the test scores over the measurement intervals. Model estimation terminated successfully for Model 1, *χ*^2^ (73) = 195.116. The RMSEA for model 1 is 0.069, 90%CI [0.058, 0.081] which implies an adequate fit. The TLI for Model 1 is 0.953 and above the value for determining a good fit for model acceptability. The Comparative fit index (CFI) for Model 1 is 0.949. The standardized root mean square residual (SRMR) is 0.068.

**Figure 3:**
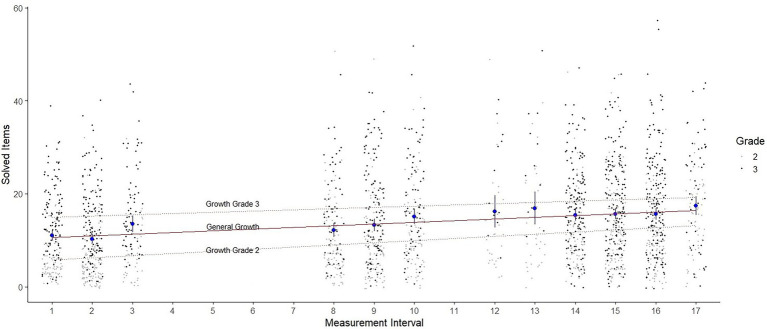
Jitter plot with the distribution of scores in the 12 LPM measurement intervals. The red line illustrates the general learning growth. The light dashed lines illustrate the growth for grade 2 and grade 3. The blue dots show the mean value as well as the confidence intervals of the respective measurement intervals for the overall sample.

The slope, as a measure of linear growth in mental addition and subtraction competence over time, is positive for Model 1 (estimate = 0.342; *SE* = 0.024; *p* < 0.001), indicating that mental computation skills have improved over the survey period (see also Figure 3). On average, the students solved roughly one more task correctly every three measurement intervals. Considering the grade level, second grade students solved roughly one more task correctly every two measurement intervals (estimate = 0.439; *SE* = 0.030; *p* < 0.001), for third grade students this was about every four measurement intervals (estimate = 0.249; *SE* = 0.033; *p* < 0.001). Data thus suggest a slightly steeper learning curve for second graders, implying faster learning.

The variance of the slope is also statistically significant for Model 1 (*p* < 0.001), indicating that learning growth did not change at the same rate for all students (see also Figure 3). Of the 222 students who completed the minimum six measurement time points required by Christ et al. (2013), 213 students (95.95%) had an individual positive slope, indicating that they exhibited learning growth over time. Positive slope values ranged from 0.001 to 1.176, indicating that some students were able to solve up to one more task per interval on average.

In the previous LGCM model (Model 1), individual change over time was indicated by intercept and slope, including only grade as covariate. In a further step, we extend the LGCM model to include group differences according to the research questions 2.2 and 2.3.

Model estimation terminated successfully for Model 2, *χ*^2^ (113) = 236.477. The RMSEA for model 2 is 0.056, 90% CI [0.046, 0.066] which implies a close to adequate fit. The TLI for model 2 is 0.952 and is also above the value for determining a good fit for the model acceptability. For Model 2, CFI is 0.952 and the SRMR is 0.057. In comparison to Model 1, the indices suggest a slightly better fit of Model 2.

The intercept of the latent learning curves in model 2 differed based on gender (estimate gender = −3.313, *p* < 0.001). Data revealed a higher intercept for males in comparison to females at the beginning of the measurement, that is male participants were able to solve approximately three tasks more correctly than females. The intercept also differed based on grade level (estimate grade level = 10.311, *p* < 0.001), indicating that third graders solved ~10 tasks more than second graders at the beginning of the measurement. Furthermore, the intercept differed based on the assignment of SEN (estimate SEN-L = −9.385, *p* < 0.001; estimate SEN-CI = −4.015, *p* = 0.009). This indicates that students with special educational needs in the area of learning solved ~9 tasks less than students without such special need, whereas students with a special need in the area of communication and interaction solved ~4 tasks less. All results are reported in Table 9.

**Table 9:** Parameter estimates for linear latent growth model (Model 2).

		Estimate	Estimate (Std. all)	*p*
*Mean*				
Intercept		3.644	0.478	0.187
Slope		1.077	3.503	≤0.001
*Variance*				
Intercept		29.758	0.512	≤0.001
Slope		0.083	0.879	≤0.001
*Covariances*				
Intercept–slope		0.531	0.338	≤0.001
*Regressions*				
Intercept	SEN-L	−9.385	−0.280	≤0.001
	SEN-CI	−4.015	−0.114	0.009
	Grade level	10.311	0.675	≤0.001
	Gender	−3.313	−0.217	≤0.001
Slope	SEN-L	−0.150	−0.111	0.139
	SEN-CI	−0.017	−0.012	0.868
	Grade level	−0.178	−0.289	≤0.001
	Gender	−0.073	−0.119	0.105

Focusing on the impact of the factors on the learning slope, only grade level led to a significantly differing learning slope (estimate = −0.178, *p* < 0.001), indicating that third graders learning was slightly slower than second graders learning. The learning slope did not significantly differ for males versus females (estimate = −0.073, *p* = 0.105) or for students with and without SEN-L (estimate = −0.150, *p* = 0.139) or with and without SEN-CI (estimate = −0.017, *p* = 0.868).

## Discussion

The present study used a newly developed LPM tool to investigate the latent learning growth curves in mental addition and subtraction of second and third graders and the influence of sociodemographic characteristics such as grade level, gender, and the assignment of SEN on these curves. Thus, this study addressed the research stages 1 and 2 outlined by Fuchs (2004) to classify LPM research, both of which are prerequisites for the valid interpretation of the gathered data regarding individual learning curves.

### LPM research at stage 1

In order to address research stage 1, we used a number of reliability and validity tests to examine the psychometric quality of the LPM tool as a static score. Correlations between sum scores of adjacent measurement intervals were strong, while sum scores of measurement intervals more distant in time showed the expected, somewhat lower correlations. As measured criterion validity, correlations between the LPM sum scores at the first and last measurement point with the arithmetic subscales of the DEMAT 2+ were moderate to strong. As expected, the correlations were lower at the last measurement time point. In this regard, it should be kept in consideration that the DEMAT 2+ reflects requirements of the mathematics curriculum of the second grade (Krajewski et al., 2020). By the end of grade 3, most students without SEN should be able to solve the items of the DEMAT 2+. Furthermore, as a measure of predictive validity, the association between the LPM sum scores at the first measurement and sum scores of the DEMAT 2+ arithmetic subscales were moderate to strong and are an indication of the important role of a mental computation in the solution of further arithmetic problems. Thus, even a single measurement in winter with the LPM tool can be a solid predictor of arithmetic performance at the end of the school year.

### LPM research at stage 2

Over and above the psychometric characteristics at stage 1, significant positive linear growth in LGCM analyses indicates that the LPM tool is sensitive to students learning (Stage 2). Both, the slopes and the variance in slopes were significant, showing that meaningful learning has occurred over the 17 measurement intervals and that students significantly differ in their learning growth. This is also reflected in the broad range of individual slope values. These findings are consistent with the results of the study by Salaschek and Souvignier (2014). In their study, they reported significant differences in learning growth in second grade students’ computation skills. In their study, second graders on average solved just under one more item per 3-week measurement interval, whereas in our study students solved one more item correctly every 4 weeks. Nevertheless, the results are only comparable to a limited extent as the LPM computation tests by Salaschek and Souvignier (2014) included tasks with all four basic arithmetic operations and reflected second-grade curriculum goals. In contrast, the LPM test employed in this study included mixed addition and subtraction tasks with varing difficulty based on the underlying DGICs. Moreover, the LPM test in this study required students to write the correct solution in a blank field, which allows a qualitative analysis of errors and eliminates guessing, the LPM computation tests by Salaschek and Souvignier (2014) were presented in a multiple-choice format.

Regarding the comparison of learning growth for weaker and stronger students, based on the continous norming approach, we observed that students in the upper percentiles have higher learning growth than students in the lower percentiles, who barely improved over the measurement intervals. This highlights prior longitudinal or crossed-lagged findings regarding the high impact of prior knowledge on future learning in mathematics (e.g., Star et al., 2009) and underlines the relevance of this research. However, analyses also highlight a positive result: Almost 96% of the students achieved an individual positive slope even though the positive slope values were relatively heterogeneous ranging from 0.001 to 1.176 (i.e., an average improvement between 0.001 and 1.176 items over the whole measurement period of 17 biweekly measurement intervals). Nonetheless, the results for the growth curves show a significant floor effect for students at the lower end of the distribution. These findings are of particular practical relevance, as it highlights the benefit of close use of LPM tools to identify learners with small or no learning growth at an early stage and provide appropriate learning support to prevent learning stagnation and ongoing mathematical difficulties. Therefore, heterogeneity in classes should be increasingly reflected in instructional decisions (e.g., Stecker et al., 2008).

In addition to these results, our study also provides information on the influence of sociodemographic characteristics such as gender and grade or the assignment of SEN on learning growth in mental computation. In our study, we found significant differences in participants’ prior achievement in favor of students in higher grades and students without SEN. Moreover, there are also differences in students learning growth development. In particular, the higher learning growth for second graders is consistent with curricular expectations and results of previous research (e.g., Selter, 2001; Benz, 2005; Karantzis, 2011). In the second grade, mental addition and subtraction with one-and two-digit numbers is curricular established and taught, whereas in the third grade, there is already an emphasis on the written computational algorithm and some students already have a fairly high level of mental computational skills. We found gender differences for the intercept, but not for the slope. Students with SEN had a significantly lower intercept value. This result is consistent with the findings that especially students with SEN-L often do not master the basic arithmetic operations taught in primary school even in secondary school (e.g., Peltenburg et al., 2012; Gebhardt et al., 2014; Rojo and Wakim, 2022).

### Limitations

There are some limitations to consider in our study. First, the COVID-19 pandemic played an important role even before the survey began (e.g., home schooling as early as the 2019/2020 school year), which implies that the results should not be interpreted free of these home schooling influences. The COVID-19 pandemic also resulted in the cancelation of scheduled measurements due to homeschooling during this survey. As a result, it was not possible to carry out all the planned measurements at all six participating schools. This resulted in a smaller than expected amount of data being available for some measurement intervals. Moreover, the observed latent learning curves may be somewhat less steep than expected with regular teaching. Thus, future longitudinal surveys will need to confirm our findings.

Second, only few students with the assignment of SEN participated in the study and they were unevenly distributed across the grades. This is mainly due to the fact that in Germany, SEN, especially SEN-L, is often not allocated until the third grade.

Third, while mental computation is an important domain of overall mathematics competence, it is also a relatively narrow focus in regard of mathematics skills. Therefore, it is not appropriate to interpret the results in such a way that they provide valid information about the overall performance in mathematics (e.g., see Christ et al., 2008).

Fourth, the influence of other important individual characteristics on mathematics performance such as working memory or language skills were not addressed in our study, although these could have an influence on task processing (e.g., Purpura and Ganley, 2014).

Fifth, our mental computation test consisted of visually administered items. Previous research (e.g., Reys et al., 1995) suggests that students’ mental computation performance may be influenced by the mode of task presentation (e.g., visually or orally). This cannot be investigated in our study as the test did not contain orally administered items.

Sixth, the results show that the tasks are suitable for measuring learning development, but do not yet cover all performance domains. In particular, more simple computation tasks are needed to more accurately measure learning development in the lower skill range in the future. For this purpose, the used DGICs can provide valuable information about the obstacles to solving tasks correctly and for the construction of easier tests that can more sensitively measure mental computation skills at the lower performance levels.

Seventh, our study does not provide information about solution strategies that students used when completing the multi-digit addition and subtraction tasks. Accordingly, no statements can be made about the adequacy and flexibility of the students’ use of solution strategies. Nevertheless, we assume that a higher sum value of correct items over time implies a more elaborate use of solution strategies.

### Future research

Future studies need to further investigate how LPM tests can be systematically used by teachers to improve the mental computation skills of their students. Identifying where differences in mental computation occur can support teachers develop appropriate educational instruction to meet the needs of individual students (e.g., Yarbrough et al., 2017). Our item design based on four DGICs will allow us to make statements that are even more concrete about areas that were specifically challenging for students, possibly pointing to student misconceptions and thus area that need specific teacher attention and support. In this regard, we will be able to offer not only a general performance score, but also differentiated scores according to the four DGICs. This allows us to provide teachers with more specific qualitative feedback on students’ mental computational performance. In the context of DGIC-focused analyses, there are several questions of relevance: A first important question would be whether the influence of DGICs changes over time (e.g., whether the DGIC necessity of crossing ten loses influence over time). For example, in order to provide tailored math instruction, for teachers it would be useful to know which hurdles in the learning process students have already successfully mastered and which they have not. Following on from this, a second important question is which students have longer-term difficulties in mastering specific hurdles. Our results show that in particular students with SEN have lower skills and less learning growth over time. A further investigation could be to examine the reasons for these performance differences and apparent stagnation of some low-achieving students, which for example might be related to insufficient knowledge or ineffective use of specific computation strategies.

Furthermore, future studies should examine trajectories in mental computation to describe how students differ in their skills and what characterizes different groups of learners. This information can both help identify students with learning difficulties in mental computation and provide trajectory-specific instructions (e.g., Salaschek et al., 2014).

Another issue arises from the construction of the parallelized tests that we used. While they were parallel in item selection based on the DGICs and should thus be comparable regarding their difficulty, there is no specific way to test this hypotheses. However, we assume, that the randomization by item category harmonize the difficulties enough to observe substantial inference.

In conclusion, we developed an LPM tool for mental computation that meets the criteria of LPM research stages 1 and 2. This lays important foundations for its future use as an LPM instrument in general as well as in regard of its use in computerized adaptive testing approaches (e.g., Frey and Seitz, 2009). However, to normalize scores that address a broader proficiency range by computerized adaptive testing, the scoring mechanism (e.g., sum scores) has to be modified and the item parameters have to be fixed. We believe that the current study is a step in this direction.

The results of our study underline the high variability of mental computation skills and illustrate that one-size-fits-all instruction is not appropriate. Instead, teachers need to obtain insight into the different learning growth curves based on LPM data and provide individualized learning offers (e.g., Hickendorff et al., 2019). Otherwise, a lack of mental computation skills can be a hurdle for future learning success in mathematics. The study provides a strong reference against which individual growth can be compared to identify struggling students in mental computation and provide targeted support based on qualitative error analysis.

## Data availability statement

The raw data supporting the conclusions of this article will be made available by the authors, without undue reservation.

## Ethics statement

Ethical review and approval was not required for the study on human participants in accordance with the local legislation and institutional requirements. Written informed consent to participate in this study was provided by the participants’ legal guardian/next of kin.

## Author contributions

SA is the primary author, conducted data collection, data preparation and analysis, created the initial version of the manuscript, and guided the further writing process. MS supported data collection, data preparation and analysis, and provided feedback in the writing process. DS provided theoretical expertise and feedback in the writing process. MG provided writing oversight and feedback in the writing process. All authors contributed to the article and approved the submitted version.

## Funding

The current research is part of the project Dortmund Profile for Inclusion-Oriented Learning and Teacher Training – DoProfiL. DoProfiL is part of the ‘Qualitätsoffensive Lehrerbildung’, a joint initiative of the Federal Government and the Länder, which aims to improve the quality of teacher training. The programme is funded by the Federal Ministry of Education and Research (Bundesministerium für Forschung und Bildung; Förderkennzeichen 01JA1930). The authors are responsible for the content of this publication.

## Conflict of interest

The authors declare that the research was conducted in the absence of any commercial or financial relationships that could be construed as a potential conflict of interest.

## Publisher’s note

All claims expressed in this article are solely those of the authors and do not necessarily represent those of their affiliated organizations, or those of the publisher, the editors and the reviewers. Any product that may be evaluated in this article, or claim that may be made by its manufacturer, is not guaranteed or endorsed by the publisher.

## Footnotes

^1^In inclusive education classes in Germany, students with and without SEN are taught together. Students with SEN sometimes have individual learning goals that do not have to correspond to the curricular goals of classmates without SEN. In special education classes, only students with SEN are taught. The learning goals can follow curricular or individual learning objectives, depending on the type of SEN.

^2^ www.levumi.de
